# An Analytic Contemplation of the Conspicuous Vicissitudes in the Histomorphology of Corpuscles of Stannius of a Freshwater Catfish* Mystus tengara* (Hamilton, 1822) due to the Exposure of ZnS Nanoparticles

**DOI:** 10.1155/2015/697053

**Published:** 2015-11-29

**Authors:** Nilanjana Chatterjee, Baibaswata Bhattacharjee

**Affiliations:** ^1^Department of Zoology, Ramananda College, Bishnupur, Bankura 722122, India; ^2^Department of Physics, Ramananda College, Bishnupur, Bankura 722122, India

## Abstract

Enhanced surface photooxidation property associated with the ZnS nanoparticles caused the reduction of dissolved oxygen content in water in a dose dependent manner, when ZnS nanoparticles of different sizes are exposed to the water in various concentrations. This property was more prominent for ZnS nanoparticles with smaller sizes.* Mystus tengara*, exposed to ZnS nanoparticles, responded to hypoxia with varied behavioural, physiological, and cellular responses in order to maintain homeostasis and organ function in an oxygen-depleted environment. The histomorphology of corpuscles of Stannius of the fish showed conspicuous vicissitudes under exposure of ZnS nanoparticles. The population of the cell type with granular cytoplasm showed significant increase at the expense of the other that consisted of agranular cytoplasm with increasing nanoparticle concentration. This can be explained as the defence mechanism of the fish against ZnS nanoparticle induced hypoxia and environmental acidification. The altering histomorphology has been studied employing an analytical approach.

## 1. Introduction

Owing to their very small sizes, nanoparticles (1–100 nm) have chemical properties that differ from those of their bulk counterparts [1]. Because of their increased reactivity, the interaction of these particles with their environment also changes [1], which is the reason for environmental concern. The increased production of nanoparticles is making it more likely that such materials will end up in watercourses with unknown consequences for aquatic life. Recently, nanoparticles have come under scrutiny for their potential to cause environmental damage [2, 3].

ZnS nanoparticle (NP) is one of such materials that can be found in the wastes of cosmetic, pharmaceutical, and rubber industries. ZnS nanoparticles are expected to exhibit some passive effects on aquatic environment by changing important physicochemical parameters of water due to its property of surface photooxidation [4]. Due to enhanced surface photooxidation property of ZnS in its nanoparticle form, the dissolved oxygen content in water is found to reduce in a dose dependent manner from its normal values, when ZnS nanoparticles of different sizes are exposed to the water in various concentrations [4–6]. This property is more prominent for ZnS nanoparticles with smaller sizes. As a result, under the exposure of ZnS NPs, the aquatic fauna of a particular habitat are forced to live in an oxygen-depleted atmosphere [4–6]. When living in a habitat with low level of dissolved oxygen, fish respond to hypoxia with varied behavioural, physiological, and cellular responses in order to maintain homeostasis and organ function in an oxygen-depleted environment [7–13].

*Mystus tengara* (Hamilton, 1822) is a species of freshwater striped catfish, found mainly in the rivers of the Indian subcontinent. This species is often confused with its closest congener* Mystus vittatus* (Bloch, 1794).* M. tengara* has a high nutritional value and it is an important target species for the small-scale fishermen. This species is a very attractive candidate for aquaculture in Southeast Asia.

The corpuscle of Stannius (CS) is present in all jawed fishes. It is a unique endocrine gland producing Stanniocalcin (STC), a homodimeric glycoprotein hormone [14]. In different species, CS either located ventrally on the surface of the kidney or scattered throughout the kidney. In bony fish, it is thought to be an important regulator of calcium and phosphate. Therefore, CS can be regarded as a very important organ in teleost fishes to maintain homeostasis and organ function in a stressful and hostile environment.

The aim of our present study is to monitor systematically the conspicuous vicissitudes in the histomorphology of corpuscles of Stannius of* M. tengara* under exposure of ZnS NPs. This will also help to realise how the growth and maturity of the fish are being altered when exposed to ZnS NPs.

## 2. Materials and Methods

### 2.1. Synthesis and Characterization of ZnS Nanoparticles (NPs)

ZnS NPs were synthesized employing simple wet chemical method as described by Chen et al. [15]. After synthesis the nanoparticles were characterized through transmission electron microscopy (TEM), particle size analysis (PSA), X-ray diffraction (XRD) study, energy dispersive X-ray (EDX) study, and X-ray photoelectron spectroscopy (XPS) study. The process of synthesis and characterization procedures of the ZnS NPs were described in detail elsewhere [5, 6]. Different characterization techniques determined indisputably that stoichiometric, spherical ZnS nanoparticles of different sizes (3 nm, 7 nm, 12 nm, and 20 nm) were synthesized under different experimental conditions of synthesis technique [6].

### 2.2. Fish Husbandry

Matured* M. tengara* specimens of both sex groups were collected from the local fishermen. Immediately after collection, fishes were kept in watertight containers containing tap water that has been allowed to stand for a few days. A good supply of necessary oxygen was provided in the container. Fishes were maintained at 25°–30°C of temperature to ensure the natural environment. The fishes were fed on natural fish foods. Small, regular supplies of food are provided.

### 2.3. Histology and Histometry

Fishes were anesthetized with a 0.3% aqueous solution of tricaine methanesulphonate (MS 222, Sigma Chemical, USA) and sacrificed. Then a longitudinal incision was made from the anal region through the ventral body wall upwards to expose the kidneys. The CS (2–4 per fish), found to be scattered along most of the length of the kidney, were expunged from the adjacent kidney tissue and immediately fixed in Bouin's fixative for 20 hours. The tissues were then dehydrated through ethanol, C_2_H_5_OH (GR, Merck, India) dried over activated molecular sieve zeolite 4A, cleared in xylene, and embedded in paraffin of melting point 56°–58°C. Thin sections of 4 *μ*m thicknesses were cut using a rotary microtome machine. The sections were stained with Delafield's Haematoxylin and Eosin stain and were observed under a compound light microscope of high resolution and eventually photographed with a digital camera attached to the microscope.

The histometry of the CS tissues was done using reticulomicrometer and ocular micrometer attached to the compound light microscope. Each measurement was done ten times and their mean value was used for further analysis.

### 2.4. Toxicity Test

*M. tengara* specimens were exposed to four concentrations (*σ* = 250, 500, 750, and 1000 *μ*g/L) of the ZnS nanoparticles of different sizes (*d* = 3 nm, 7 nm, 12 nm, and 20 nm) throughout the early growth phase (December-January) to observe its effect during the late growth phase (February). The histomorphology of the CS cells has been studied during the growth phase (December–February), so that the influence of 17*β*-estradiol [16] (synthesized mainly during the vitellogenic phase from April to August) upon the CS could be minimized. Electronic lab meters with accuracy up to one decimal point were used to measure the dissolved oxygen content and pH of the water.

### 2.5. Statistical Analysis and Curve Fitting

All data were expressed as means ± SE. One-way analysis of variance was run to compare the differences between groups treated under different experimental conditions and control groups. Differences were considered statistically significant when *P* < 0.05. Pearson's correlation coefficients (*r*) were calculated to determine the correlation, if any, between different histometric parameters and nanoparticle concentrations at a significance level of 5%. Negative *r* values prefixed by negative (−) sign and positive values without any prefix are used in the paper. Curve fitting to the experimentally obtained data was done using Origin 9.

## 3. Results and Discussions

### 3.1. ZnS NP Induced Hypoxia and Environmental Acidification

In the present study, the dissolved oxygen content in water (*D*_O_2__) was measured to be 13.2 mg/L at 15°C before any nanoparticle was introduced in it. This value was found to decrease both with increasing nanoparticle concentration and with nanoparticle exposure time in water at the same temperature. The value of dissolved oxygen content in water reached to a number as low as 5.9 mg/L for nanoparticles of size 3 nm at a concentration of 1000 *μ*g/L and exposure time of 6 days.

**Figure 1:**
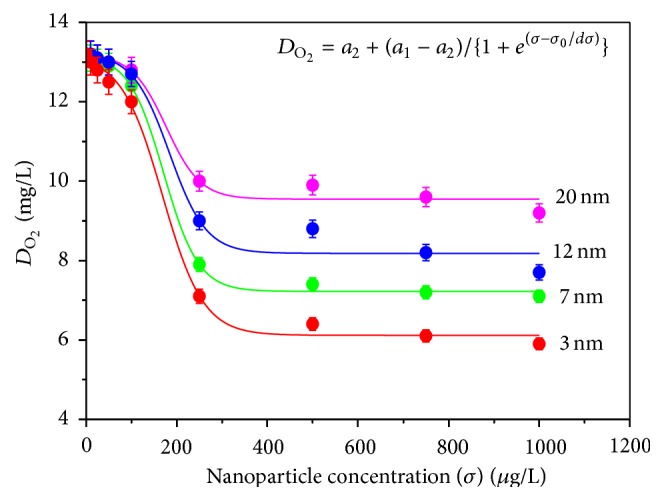
Variation of dissolved oxygen content (*D*_O_2__) in water due to exposure of ZnS nanoparticles of different sizes with increasing nanoparticle concentration (*σ*) for a fixed exposure time (*t* = 6 days).

Figure 1 shows the variation of dissolved oxygen content (*D*_O_2__) in water due to exposure of ZnS nanoparticles of different sizes (*d*) with increasing nanoparticle concentration (*σ*) for a fixed exposure time (*t* = 6 days). It is clear from the data that a threshold concentration exists for *σ* value in between 150 *μ*g/L and 200 *μ*g/L for each experimental condition. Beyond this value of ZnS nanoparticle concentration, a sudden change in *D*_O_2__ values can be clearly noticed from the figure. The data in Figure 1 are fitted well with the Boltzmann function depicted by the equation

(1) $$\begin{array}{c} D_{O_{2}}=a_{2}+\frac{\left(a_{1}-a_{2}\right)}{1+e^{\left(\sigma -\sigma_{0}\right)/d\sigma }}, \end{array}$$

where *a*_1_, *a*_2_ are constants for a particular experimental condition and *σ*_0_ corresponds to the threshold concentration for that specific trial. Examining the fitting parameters it can be inferred definitely that the particles with smaller sizes correspond to the lower value (for *d* = 20 nm, *σ*_0_ = 184.05 *μ*g/L; *d* = 12 nm, *σ*_0_ = 176.57 *μ*g/L; *d* = 7 nm, *σ*_0_ = 171.17 *μ*g/L; and *d* = 3 nm, *σ*_0_ = 168.40 *μ*g/L) of threshold concentration.

**Figure 2:**
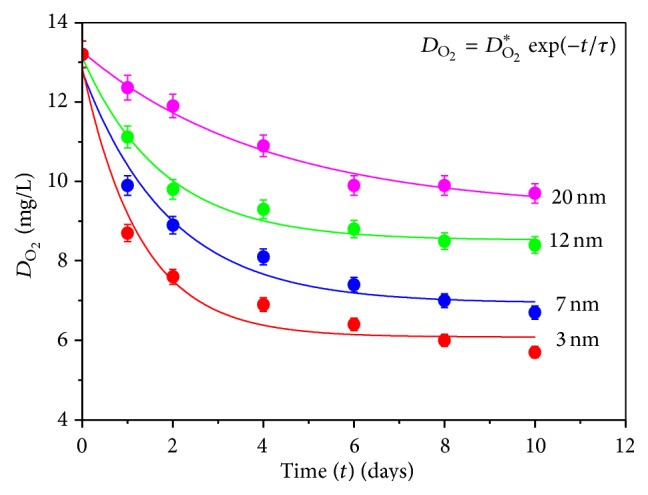
Variation of dissolved oxygen content (*D*_O_2__) in water due to exposure of ZnS nanoparticles of different sizes with increasing exposure time (*t*) for a fixed nanoparticle concentration (*σ* = 500 *μ*g/L).

Figure 2 shows the variation of dissolved oxygen content (*D*_O_2__) in water due to exposure of ZnS NPs of different sizes with increasing exposure time (*t*) for a fixed nanoparticle concentration (*σ* = 500 *μ*g/L). The data are fitted well with the first-order exponential decay curve expressed by the equation

(2) $$\begin{array}{c} D_{O_{2}}=D_{O_{2}}^{*}\exp\left(\;-\frac{t}{\tau }\;\right), \end{array}$$

where *D*_O_2__^*∗*^ is a constant for a fixed trial and *τ* determines the slope of the curve. Fitting parameters again revealed greater rate of change in *D*_O_2__ value for smaller ZnS nanoparticles.

The photooxidation of the surface of ZnS NPs using the dissolved oxygen of water under sunlight and consequent reduction of dissolved oxygen content in water has been confirmed from detailed study of S 2p core level X-ray photoelectron spectra of ZnS nanoparticles after different time of exposures [6]. During the surface photooxidation process of ZnS NPs, The S atoms exposed to the ZnS surface got oxidized and an increase in concentration of chemisorbed SO_2_ at ZnS surface with increasing exposure time was observed in the samples [6]. The oxide leaves the surface as a molecular species (SO_2_), leaving Zn and a freshly exposed layer of ZnS behind. Water may dissolve a part of the SO_2_ released in the process causing reduction in the pH value of the water [4]. Subsequently under the exposure of ZnS NPs, the aquatic fauna of that particular habitat were forced to live in an oxygen-depleted and acidified atmosphere [4–6].

In the present study, the pH value of water was found to decrease when exposed to ZnS NPs in a dose dependent manner for a fixed exposure time of 6 days. In controlled condition the pH value of the water used in this experiment was measured to be 7.6. This value was found to decrease both with increasing nanoparticle concentration and with nanoparticle exposure time in water for a fixed nanoparticle size. The rate of reduction in pH value was found to be higher for the nanoparticles with smaller sizes. In our experiment, the pH value of water dwindled down to 6.2 for nanoparticle concentration (*σ*) of 1000 *μ*g/L with size (*d*) 3 nm and exposure time (*t*) of 6 days. Reduction of water pH and consequent acidification of the environment finally lead the fishes to metabolic acidosis.

**Figure 3:**
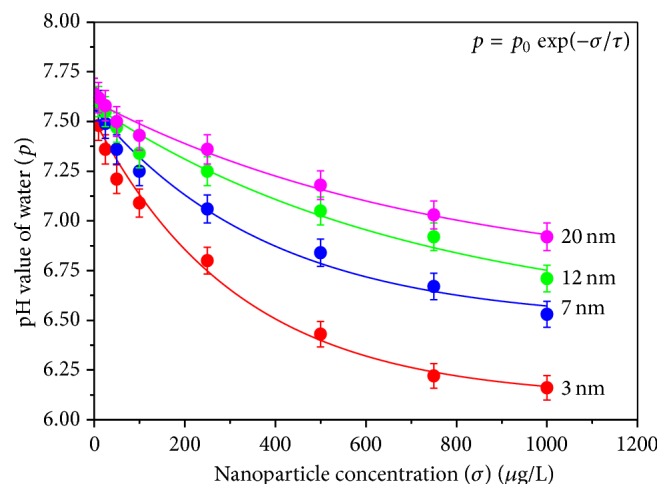
Variation of pH value of water due to exposure of ZnS nanoparticles of different sizes with increasing nanoparticle concentration (*σ*) for a fixed exposure time (*t* = 6 days).

Figure 3 shows the variation of pH value of water (*p*) due to exposure of ZnS nanoparticles of different sizes with increasing nanoparticle concentration (*σ*) for a fixed exposure time (*t* = 6 days). The data shown in this figure are fitted well with the first-order exponential decay curve expressed by the equation

(3) $$\begin{array}{c} p=p_{0}\exp\left(\;-\frac{\sigma }{\tau }\;\right), \end{array}$$

where *p*_0_ is a constant for a fixed trial and *τ* determines the slope of the curve. Study on the fitting parameters exposed greater rate of change in pH value due to exposure of smaller ZnS nanoparticles.

After the exposure of the ZnS NPs in the water, the Zn/S ratio in the nanoparticles was found to rise over that of the stoichiometric value of the freshly prepared samples confirming the loss of S from the surface of the nanoparticles. Surfaces of the ZnS NPs, exposed to water and light, were thus effectively destroyed by the redox cycles and resulted in the reduction of the dissolved oxygen content and pH value of water.

Alterations in physicochemical parameters of water were found to be more prominent for ZnS NPs with smaller sizes. This observation could be explained by the fact that smaller particle size culminated higher surface to volume ratio of the nanoparticles present in the water. Therefore, ZnS NPs having smaller sizes offered greater surface area, making the particles more sensitive to surface photooxidation process. This leads to a faster deficit in dissolved oxygen content and reduction in pH values when exposed to water compared to the samples having larger particle sizes.

### 3.2. Histology of Corpuscles of Stannius (CS)

**Figure 4:**
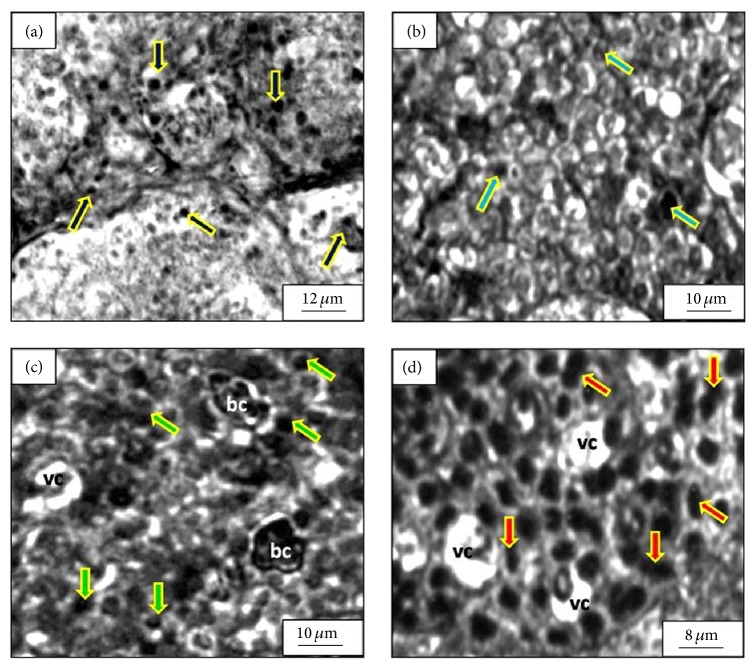
Transverse histological section of the corpuscles of Stannius (CS) in* Mystus tengara*: (a) under controlled condition few of the cells show large nuclei with agranular cytoplasmic contents (solid arrow), (b) under ZnS NP exposure (250 *μ*g/L, *d* = 3 nm, and *t* = 60 days) amongst most of the cells with agranular cytoplasm few cells with darkly stained nuclei and granulated cytoplasm (solid arrow) are also visible, (c) under ZnS NP exposure (500 *μ*g/L, *d* = 3 nm, and *t* = 60 days) most of the cells have dense granulated cytoplasm (solid arrows) and the nuclear volume of the cells is found to increase along with their condensed status (shown by deeply stained large nuclei), and (d) under ZnS NP exposure (1000 *μ*g/L, *d* = 3 nm, and *t* = 60 days) most of the cells have been found with dense granulated cytoplasm with large elongated and dumbbell shaped (solid arrows) hyperactive nuclei with highly condensed chromatin mass (deeply stained). Some large vacuoles are not uncommon. (bc: blood cells, vc: vacuoles.)

Figures 4(a)–4(d) show the histology of corpuscles of Stannius (CS) under controlled and different experimental conditions in the late growth phase of February. Under controlled condition (Figure 4(a)), CS cells are found to be closely packed and regularly arranged in different lobules depicting the normal and healthy status of the CS tissues. In this condition, the cells are found to be polygonal to round in shape having mean diameter of 7.8 ± 1.2 *μ*m. Predominant cell types are characterized by lightly stained centrally located nucleus along with agranular cytoplasm. However, a few cells are noticed with conspicuously large nuclei and chromophobic cytoplasm.

Salient changes in the histology of CS can be observed owing to the exposure of ZnS NPs of different concentrations. Under the exposure of ZnS NP of 250 *μ*g/L (Figure 4(b)) having size of 3 nm, a few cells with darkly stained nuclei and granulated cytoplasm are also visible from the micrograph within most of the cells with agranular cytoplasm. Though the cell size was found to be almost the same as the controlled condition, the ratio of nuclear to cellular volume was found to increase significantly (*P* < 0.005).

Under the exposure of ZnS NP of 500 *μ*g/L (Figure 4(c)) having size of 3 nm, the number density of cells having dense granulated cytoplasm is found to rise at the expense of the cells having agranular cytoplasm making those cells the predominant cell type. The nuclear volumes of the cells continue to increase along with their condensed status. Onset of vacuolization within the tissue layout can be clearly observed from the micrographs.

Under the exposure of ZnS NP of 1000 *μ*g/L (Figure 4(d)) having size of 3 nm, most of the cells have been found with dense granulated cytoplasm. These cells portray large elongated and dumbbell shaped hyperactive nuclei with highly condensed chromatin mass. The ratio of nuclear to cellular volume was found to reach almost unity. Presence of large vacuoles is common in the tissue layout of CS under this experimental condition.

Despite extensive research on fish CS [17–24], the debate concerning its cellular population is still open. Two structurally different cell types have been reported in the CS of several teleosts [17–21]. On the other hand, researchers also have noticed only one cell type in the CS of some other teleosts [22–24]. It is still unclear whether these represent different physiological conditions of a single cell type or depict functionally different cell types. There is evidence that the two cell types come from a single lineage, whereby their histological differences may reflect different stages of development or different states of activity [25–27]. It has even been suggested that type 2 cells may represent type 1 cells that are undergoing programmed cell death or apoptosis [28]. In the present study, the two types of cells can be inferred as the different states of activity of a single cell type. The population of the cell type with granular cytoplasm showed significant increase (*P* < 0.005) at the expense of the other cell type as a defence mechanism against the stress produced by ZnS NPs through altering the physicochemical parameters of the water.

In teleosts, the stress hormone cortisol and STC are both involved in the regulation of ion balance [29]. Pierson et al. [29] studied the effect of the stress on STC secretion and reported an elevated level of STC in freshwater rainbow trout (*Oncorhynchus mykiss*) with increasing stress signals. Their study suggested a broader function of STC in addition to its classical hypocalcaemic action. In our present study, ZnS NP was found to create hypoxia and acidification of the water in which it is exposed. These hostile conditions are supposed to induce stress signals which were responsible for disturbing the ion balance in* M. tengara*. Gradual increase in the number of cells having hyperactive nuclei and granular cytoplasm in CS with increasing ZnS NP concentration can be attributed to the onset of elevated level of secretion of STC as a response to the enhancement of external stress factors.

### 3.3. Histometry of Corpuscles of Stannius (CS)

The ratio percentage of number density of cells with agranular cytoplasm (*n*_*a*_) to the cells with granular cytoplasm (*n*_*g*_) in a particular potion of the CS histological tissue layout can be defined as

(4) $$\begin{array}{c} \xi =\frac{n_{a}}{\left(n_{g}+n_{a}\right)}\times 100\%. \end{array}$$

**Figure 5:**
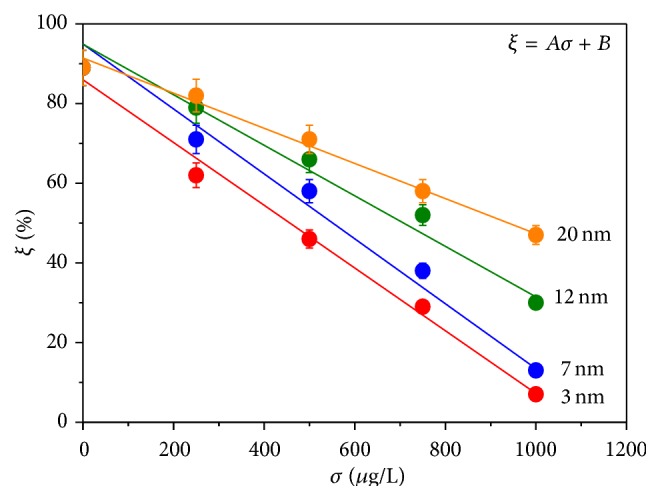
Variation of the ratio percentage of the number density of cells in CS (*ξ*) of* Mystus tengara *with increasing ZnS nanoparticle concentration (*σ*) for a fixed exposure time (*t* = 60 days) keeping nanoparticle size (*d*) as a parameter.

*ξ* value is found to decrease with increasing NP concentration (*σ*) and shows strong negative correlation with *σ*.  Figure 5 shows the family of curves depicting the vicissitudes of *ξ* against *σ* for a constant exposure time (*t* = 60 days) taking nanoparticle size *d* as a parameter. The experimental data are fitted well with straight lines represented by the equation

(5) $$\begin{array}{c} \xi =A\sigma +B, \end{array}$$

where *A*'s represent the slopes of the straight lines and *B* the intercepts of the lines on the *ξ* axis. Higher value of *A* indicates greater dependence of *ξ* on *σ*.  Table 1 shows the fitting parameters to the experimental data and the correlation coefficients. It is clearly noticed from the table that the lines became steeper with decreasing particle size indicating stronger effect for smaller particles. It can also be detected from the table that there is strong negative correlation between *ξ* and *σ* for any of the particle sizes.

The ratio of nuclear volume (*V*_*n*_) to cellular volume (*V*_*c*_) in CS tissue layout can be defined as

(6) $$\begin{array}{c} \zeta =\frac{V_{n}}{V_{c}}. \end{array}$$

**Figure 6:**
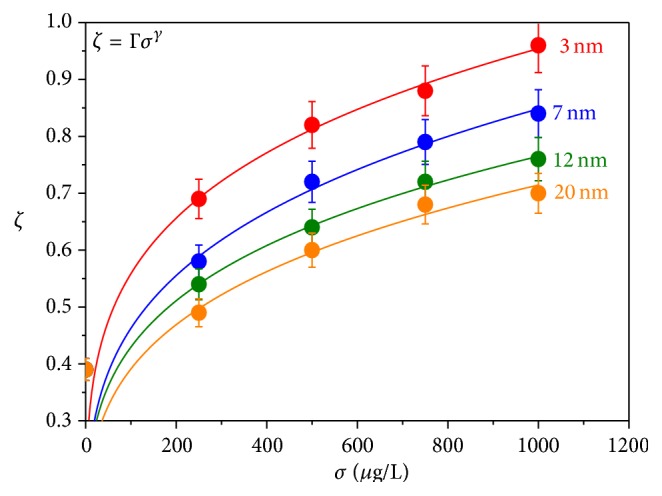
Variation of the ratio of nuclear volume (*V*_*n*_) to cellular volume (*V*_*c*_) in CS tissue layout (*ζ*) of* Mystus tengara* with increasing ZnS nanoparticle concentration (*σ*) for a fixed exposure time (*t* = 60 days) keeping nanoparticle size (*d*) as a parameter.

Nuclear and cellular volumes were calculated assuming the nuclei and cells as ellipsoids. The *ζ* value shows strong positive correlation with *σ*.  Figure 6 shows the family of curves depicting the vicissitudes of *ζ* against *σ* for a constant exposure time (*t* = 60 days) taking nanoparticle size *d* as a parameter. The experimental data are fitted well with allometric function represented by the equation

(7) $$\begin{array}{c} \zeta =\Gamma \sigma^{\gamma }, \end{array}$$

where Γ's are a coefficient different for different set of experimental points and *γ* is an exponent. Higher value of *γ* indicates greater dependence of *ζ* on *σ*.  Table 2 shows the fitting parameters to the experimental data and the correlation coefficients. It can be seen from the table that *γ* value increases with decreasing particle size expressing sturdier influence for smaller particles. It can also be demonstrated from Table 2 that there is strong positive correlation between *ζ* and *σ* for any of the particle sizes.

## 4. Conclusion

The histomorphology of the corpuscles of Stannius (CS) of a freshwater catfish* Mystus tengara* showed conspicuous vicissitudes under the exposure of ZnS nanoparticles (NPs). As a defence mechanism against the stress induced by ZnS NPs, the population of the cell type with granular cytoplasm showed significant increase at the expense of the other cell type. The cell density with hyperactive nuclei was found to increase remarkably. This can be associated with the onset of the elevated level of Stanniocalcin (STC) secretion as a response to the enhancement of the external stress factors. An analytic contemplation was employed to describe the whole process quantitatively.

## Figures and Tables

**Table 1:** Parameters used for fitting of the straight lines to the experimental data showing the variation of the ratio percentage of the number density of cells in CS (*ξ*) of *Mystus tengara* with increasing ZnS nanoparticle concentration (*σ*) for a fixed exposure time (*t* = 60 days) keeping nanoparticle size (*d*) as a parameter and the correlation coefficients between *ξ* and *σ* for a fixed *d*.

Nanoparticle size (*d*) (nm)	*A* (L/*μ*g)	*B* (%)	*R* ^2^	Pearson correlation coefficient (*r*)
3	−0.081	86	0.989	−0.996
7	−0.082	97.974	0.984	−0.994
12	−0.063	94.925	0.972	−0.989
20	−0.044	91.458	0.992	−0.979

**Table 2:** Parameters used for fitting of the curves to the experimental data showing the variation of the ratio of nuclear volume (*V*_*n*_) to cellular volume (*V*_*c*_) in CS tissue layout (*ζ*) of *Mystus tengara* with increasing ZnS nanoparticle concentration (*σ*) for a fixed exposure time (*t* = 60 days) keeping nanoparticle size (*d*) as a parameter and the correlation coefficients between *ζ* and *σ* for a fixed *d*.

Nanoparticle size (*d*) (nm)	Γ (L/*μ*g)^*γ*^	*γ*	Reduced *χ* ^2^	*R* ^2^	Pearson correlation coefficient (*r*)
3	0.192	0.263	1.260 × 10^−4^	0.990	0.943
7	0.138	0.261	1.676 × 10^−4^	0.987	0.966
12	0.136	0.250	5.086 × 10^−5^	0.995	0.976
20	0.117	0.232	2.884 × 10^−4^	0.968	0.977
